# Carotid Web as a Rare Cause of Stroke: A Case Report

**DOI:** 10.1155/carm/6341586

**Published:** 2026-04-11

**Authors:** Jovana Ivanovic, Tamara Svabic Medjedovic, Dragoslav Nestorovic, Ivan Vukasinovic, Vladimir Cvetic, Marko Dragas, Dejana Jovanovic, Predrag Stanarcevic

**Affiliations:** ^1^ Neurology Clinic, University Clinical Center of Serbia, Belgrade, Serbia, kcs.ac.rs; ^2^ Faculty of Medicine, University of Belgrade, Belgrade, Serbia, bg.ac.rs; ^3^ Center for Radiology and MRI, University Clinical Center of Serbia, Belgrade, Serbia, kcs.ac.rs; ^4^ Vascular Surgery Clinic, University Clinical Center of Serbia, Belgrade, Serbia, kcs.ac.rs

**Keywords:** acute ischemic stroke, carotid web, endovascular, stenting

## Abstract

A carotid web is a rare, focal vascular malformation at the posterior wall of the carotid bulb, which can be associated with acute ischemic stroke, especially in young patients. Also, a carotid web is associated with a high risk of stroke recurrence, despite optimal antithrombotic therapy. Our patient is a 41‐year‐old, previously healthy female, with a symptomatic carotid web on the right carotid artery, which led to acute embolic ischemic lesions in the right insular and frontal juxtacortical regions. She had recurrent episodes of mild left‐sided hemiparesis and dysarthria, with complete spontaneous recovery. Extensive diagnostic procedures did not confirm any other cause of stroke except a symptomatic carotid web. She underwent stent placement, with no new vascular events, a normal neurological examination, and maintaining stent patency 1 year after the stroke onset. A carotid web is an important cause of stroke in young patients, potentially preventable by endovascular stent placement, alongside the best medical treatment.

## 1. Introduction

A carotid web is recently recognized as an important cause for cryptogenic stroke, and it presents as a focal nonatherosclerotic vascular intraluminal shelf‐like defect at the posterior margin of the carotid bulb and could be associated with fibromuscular dysplasia (FMD) [1]. Also, a carotid web can occur isolated, with possible influence of genetic factors, vessel wall damage, or hormone abnormalities [2]. Carotid web represents a rare cause of embolic stroke of undetermined source (ESUS), particularly in young patients, [2, 3]. Having in mind that these patients typically have young stroke onset, extensive diagnostic algorithms should be performed including brain and vessel imaging, extracranial and intracranial ultrasonography, cardiac examination, and detailed serologic analyses to rule out the influence of infectious, immune‐mediated or prothrombotic factors. This local vessel abnormality develops as a focal intimal hyperplasia, leading to fibrosis, degeneration, and protrusion into the lumen of the carotid artery [2]. The approximate prevalence of carotid web is about 1.2% and leads to recurrent ischemic stroke in 20%–32% of patients, regardless of antithrombotic therapy [1, 4]. There is no clear evidence on whether a carotid web is a congenital or acquired malformation with possible influence of both genetic and environmental factors [5]. Potential pathophysiological mechanisms for stroke onset include altered and turbulent blood flow that results in stasis, thrombosis, and distal embolization [5]. There are no randomized controlled trials regarding optimal therapeutic approaches in these patients, who are usually treated with antiplatelet drugs [3]. The case of our young patient, whose favorable recovery resulted from prompt diagnosis and management, underscores the carotid web as an underrecognized but potentially preventable cause of stroke.

## 2. Case Presentation

**FIGURE 1:**
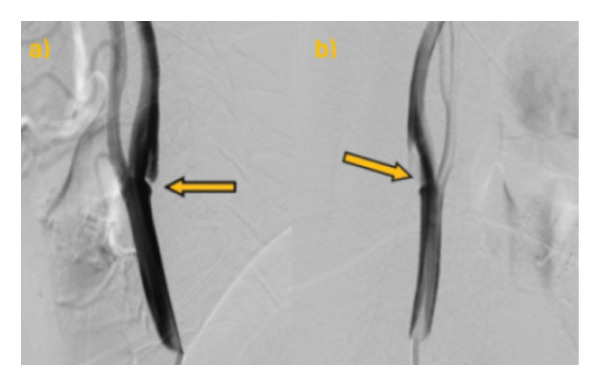
Digital subtraction angiography shows (a) a symptomatic carotid web on the right ICA presented as a linear membranous structure with lumen reduction, shown with an arrow and (b) stagnation of contrast in the carotid web shown with an arrow.

**FIGURE 2:**
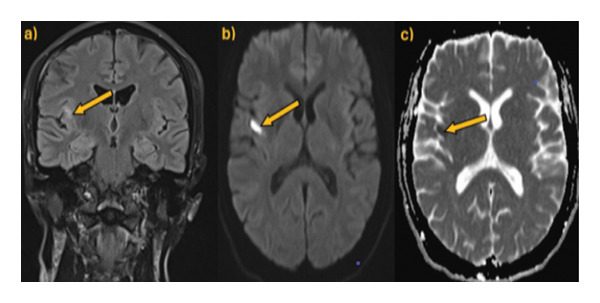
Brain MRI showed acute embolic ischemic lesions in the right insular and frontal juxtacortical regions, pointed with arrows: (a) hyperintensity on axial FLAIR, (b) hyperintensity on axial DWI, and (c) hypointensity on the axial ADC map.

**FIGURE 3:**
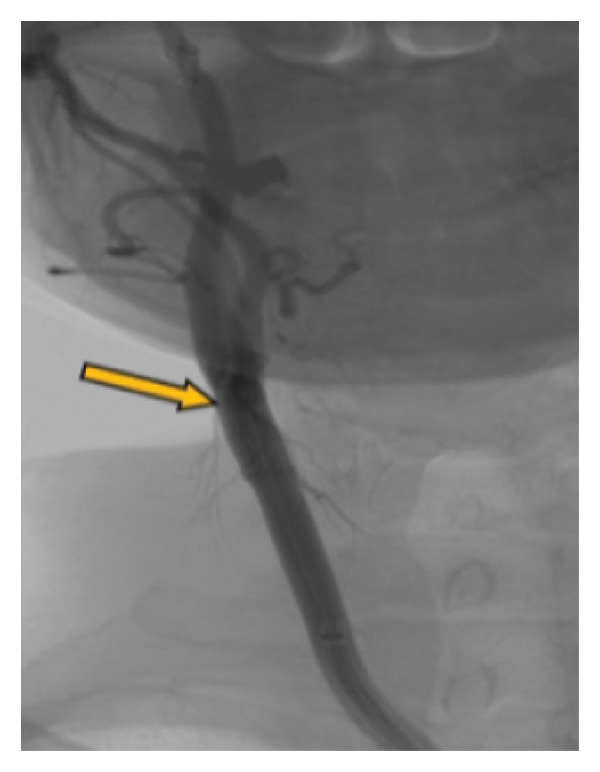
Digital subtraction angiography after the stent placement with flattening and restoring of a smooth lumen shown with an arrow.

**FIGURE 4:**
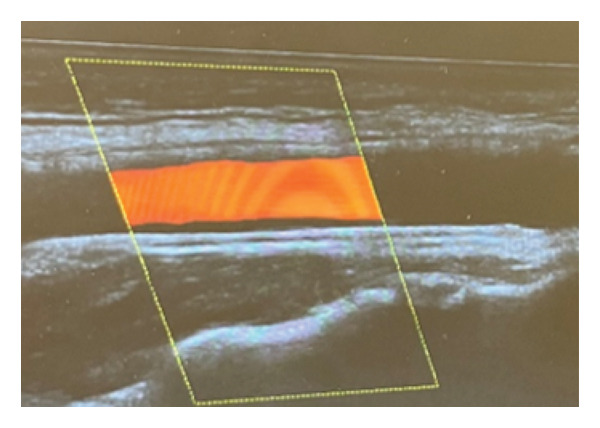
Follow‐up carotid duplex ultrasonography 1 year after the procedure showed stent patency.

Our patient is a previously healthy, 41‐year‐old female smoker with four transient episodes of mild left‐sided hemiparesis and dysarthria. Apart from smoking, there were no other known vascular risk factors including oral contraceptive use. All episodes occurred on the day of admission and recurred within 24 h. Each episode lasted about 2–3 min, with complete spontaneous resolution. On admission to our department, physical and neurological examinations were normal. Arterial pressure was 135/95 mmHg, and the ABCD2 score [6] was 3. The brain CT revealed normal findings. However, the CT angiography of intracranial and extracranial arteries showed the presence of a carotid web on the posterior wall of the right internal carotid artery. However, carotid Doppler ultrasound appeared within physiological ranges, and the transcranial Doppler ultrasound found high flow velocity in the right cerebral medial artery. Finally, we performed digital subtraction angiography, which confirmed the presence of a carotid web with distal flow turbulence and without intracranial vessel occlusion (Figure 1). Brain MRI showed acute embolic ischemic lesions in the right insular and frontal juxtacortical regions, without silent infarctions (Figure 2). Laboratory analyses confirmed hyperlipidemia (total cholesterol 6.23 mmol/L, LDL 4.02 mmol/L, and triglycerides 2.63 mmol/L) and hyperhomocysteinemia (19 μmol/L). Furthermore, analyses of the blood coagulation panel (antithrombin, prothrombin, protein C, Factor II, Factor V, Factor VIII, Factor XII, Factor XIII, the activated protein C resistance test, lupus anticoagulant, anticardiolipin, and beta‐2 glycoprotein antibodies), immunology (antinuclear and antiphospholipid antibodies), the presence of inheritable coagulopathies (Factor II G20210A, Factor V Leiden, methylenetetrahydrofolate reductase, and plasminogen activator inhibitor 1 mutations), virology, and venereal disease were made, with all results normal or negative. Transthoracic echocardiography and Holter ECG monitoring for 24 h showed no abnormalities. As our patient had a minor stroke, dual antiplatelet therapy (aspirin 100 mg/day and clopidogrel 75 mg/day) and high‐dose statin therapy (atorvastatin 80 mg/day) were immediately started on admission. After excluding other possible causes of stroke, on the eighth day after the stroke onset, the patient underwent endovascular stent placement (Figure 3). After the procedure, she had no new neurological manifestations and neurological deficits. She was discharged with dual antiplatelet therapy (aspirin 100 mg + clopidogrel 75 mg) and high‐dose statin therapy (atorvastatin 80 mg). At the last follow‐up, 1 year after the stroke onset, the patient had no new vascular events, with a normal neurological examination and maintained stent patency (Figure 4). The patient continued aspirin antiplatelet monotherapy and atorvastatin 20 mg daily.

## 3. Discussion

We presented a young female patient, with no other vascular risk factors except mixed hyperlipidemia and active smoking, with acute ischemic stroke and a symptomatic carotid web. It is considered that this vascular malformation is a variant of focal intimal atypical FMD, which involves intimal layer, while classical FMD is widespread and affects the media and adventitia [7]. Authors from Atlanta included in their study 66 patients with carotid web and showed that only 9% of patients had coexistent signs of classic FMD [7]. Our patient also had no signs of classic FMD of major cervical arteries, without a positive family history and oral contraceptive intake, although she has vascular risk factors that could be associated with the occurrence of this carotid abnormality. Zhang et al. published a systemic literature review and showed that 17% of affected patients were active smokers, and 16% of them had hyperlipidemia [8], which was also present in our patient. Carotid web is usually diagnosed by CT angiography of the head and neck, which can show a low‐density shadow with the appearance of a thin line in the transverse axial plane and a localized membrane‐filling defect in the sagittal plane, but this method can also be overlooked or misinterpreted as an atherosclerotic plaque or a dissection [2]. The incidence of this misdiagnosis is not estimated, but, on the other hand, it is shown that patients with a carotid web had a 5.41 risk ratio for developing carotid artery dissection, especially in the C1 segment [9]. DSA is the gold standard for diagnosis and can delineate the carotid web as a linear filling defect along the carotid artery wall or a shelf‐like filling defect located in the internal carotid artery bulb with continued contrast pooling in the distal part in the venous phase [2]. In our patient, CT angiography and DSA confirmed the presence of a carotid web, without affecting the intracranial arteries, although she had high flow velocity on transcranial Doppler ultrasonography. On the other hand, neurosonology can also help to establish the accurate diagnosis, although authors from France showed in their study that B‐mode was positive in 79% of patients in the longitudinal view, but the axial plane was positive in only 38% of them and concluded that microflow imaging could lead to higher sensitivity [10].

Ipsilateral intracranial occlusion is also a possible complication. The French CAROWEB registry showed a very high incidence of ipsilateral intracranial occlusion (71.8%), mainly the middle cerebral artery (53.8%), probably due to artery‐to‐artery embolism [4]. Also, intimal abnormality causes significant flow changes in the carotid bifurcation, which could be diminished after stenting or carotid endarterectomy [3]. DSA performed in our patient showed flow turbulence distal from the carotid web, which probably led to distal artery‐to‐artery embolization. There are no randomized clinical trials that would give us guidelines for the treatment of this group of patients. Due to the high risk of recurrent ischemic events despite the use of antithrombotic therapy, carotid endarterectomy and stenting appear to be the best therapeutic options for these patients [11]. Mutlon et al. presented 16 patients with symptomatic carotid web, of whom 13 were treated by open carotid surgery and 3 with carotid stenting [12]. All of them were without any ischemic event until follow‐up more than 1 year after the intervention [12]. Another cohort of patients was described by Canadian authors, who included in the study 14 patients with carotid web and stent implantation with the same outcome [13]. Recently published data from Khan et al. also showed that both carotid endarterectomy and carotid artery stenting were equally effective with substantial variability in management practices among neurointerventionalists and vascular neurologists [14], but a meta‐analysis published by the same author confirmed a strong preference for carotid artery stenting [15]. Our young patient, without atherosclerosis, also underwent endovascular stent placement as a preferred procedure, without any neurological or other adverse events 1 year after the stroke onset.

In conclusion, carotid web is a rare but underestimated cause of recurrent ischemic stroke, especially in young patients, potentially preventable by endovascular stent placement and the best medical management.

## Author Contributions

All authors have equal contributions.

## Funding

The authors received no financial support for the research, authorship, and/or publication of this article.

## Consent

Written consent was obtained for publication of the case along with accompanying images.

## Conflicts of Interest

The authors declare no conflicts of interest.

## Data Availability

The data supporting the findings of this case report (including images, clinical records, raw data, and metadata) are available from the authors and can be provided to the journal and reviewers upon reasonable request.
